# Evaluation of the Accuracy of Four Digital Methods by Linear and Volumetric Analysis of Dental Impressions

**DOI:** 10.3390/ma12121958

**Published:** 2019-06-18

**Authors:** Stefano Pagano, Michele Moretti, Roberto Marsili, Alessandro Ricci, Giancarlo Barraco, Stefano Cianetti

**Affiliations:** 1 Department of Biomedical and Surgical Sciences, Odontostomatological University Centre, University of Perugia, 06128 Perugia, Italy; stefano.pagano@unipg.it (S.P.); misure@unipg.it (G.B.); stefano.cianetti@unipg.it (S.C.); 2 Department of Engineering, University of Perugia, via G. Duranti, 93 06128 Perugia, Italy; michele.moretti@unipg.it; 3 3DIFIC, 06128 Perugia, Italy; alessandro.ricci@edific.it

**Keywords:** intraoral scanners, volumetric error distribution, point cloud registration

## Abstract

The quality of dental arch impression has a substantial role in the precision of the intervention. It is traditionally acquired with resins that solidify when in contact with the air. Compared to that method, digital impression gives great advantages and, together with three-dimensional (3D) digitization devices, allows a simplification of the digital impression process. The growing adoption of such systems by a large number of dental clinics determines the need for an in-depth evaluation of the accuracy and the precision of the different systems. The aim of this work is to define a methodology for the evaluation of the accuracy and precision of 3D intraoral and desktop scanning systems, by using volumetric and linear methods. The replica of a tooth was realized with zirconium; afterward, high-accuracy point clouds of the master model were acquired by a coordinate measurement machine (CMM). In this way, the dimensions of the replica were accurately known. An intraoral scanner (I) and three desktops (D1, D2, D3) were then used to scan the replica. The geometry resulting from the CMM was compared with the ones derived from the scanners, using two different commercial programs (Geomagic and 3-Matic) and a custom-developed algorithm (MATLAB). Geomagic showed the mean values to be in a range from 0.0286 mm (D1) to 0.1654 mm (I), while 3-Matic showed mean values from −0.0396 mm (D1) to 0.1303 mm (I). MATLAB results ranged from 0.00014 mm (D1) to 0.00049 mm (D2). The probability distributions of the volumetric error of the measurements obtained with the different scanners allow a direct comparison of their performances. For the results given by our study, the volumetric approach that we adopted appears to be an excellent system of analysis.

## 1. Introduction

In the last few years, prosthetic technology was characterized by very important changes in techniques and materials with the introduction of zirconium and lithium disilicate, and intraoral impression techniques [1,2,3,4]. In particular, in the last ten years, the introduction of Computer Aided Design (CAD) and Computer Aided Manufacturing (CAM) systems in dentistry led to several advantages for both clinicians and patients. An important step in prosthetic rehabilitation is represented by a good imprint of the dental arch/dental area, able to accurately replicate the morphology of the oral cavity. In this way, it is possible to obtain a good cast model used by technicians to realize the final rehabilitation and to clearly explain to patients the clinical procedure [4]. The traditional imprint technique requires silicon materials (obtained by addition and condensation polymerization) in single or double imprinting techniques used with knitted cords. In this way, it is possible to replicate the clinical tooth preparations with high precision, as widely shown in the literature [5,6,7,8,9,10,11,12,13,14]. However, some critical aspects about the accuracy of the traditional imprint still remain and are related to clinicians (e.g., tray choice, imprinting technique, material adhesion to the tray, use of knitted cords) [15,16], materials (e.g., hydrophilic properties, thermal expansion coefficient, syneresis, blood or saliva presence), and laboratory (e.g., humidity, temperature [8], preservation of materials, elapsed time between imprint and cast model, type of gypsum used, techniques used to obtain the study model). Moreover, the traditional imprint is generally not well accepted by patients sensitive to material taste and prone to nausea and general oral discomfort [10,17]. On the other hand, starting from the 1980s, the introduction of CAD/CAM technology in dentistry represented a revolution in prosthetic dentistry with a predicted high diffusion in the upcoming decades for different clinical situations [18]. CAD/CAM systems are generally divided into three groups: digital impression capture systems, prosthetic design software (CAD), and milling systems (CAM) [18,19]. The introduction of digital techniques shows several advantages such as the possibility to visualize in any moment the three-dimensional (3D) files, to easily modify the file multiple times for simulating different situations and, consequently, to plan the most appropriate rehabilitation. The 3D models can also be quickly shared between clinicians and technicians in every moment; finally, the imaging helps to promptly identify critical zones (e.g., reduced space, presence of undercut zones, etc.) after tooth preparation. Further advantages are the absence of impression distortion due to bad material conservation and higher patient acceptance [20,21,22,23,24,25,26]. In addition to prosthetic applications, digital technology is developing in implant surgery, as well as endodontic and orthodontic measurements [21,23,27,28,29,30].

Some different aspects have to be observed in the clinician’s choice of CAD/CAM systems: accuracy, precision, clinic operativity (scan speed, matting, tip dimension especially for molars, color images), open or closed systems (the faculty to have not only an owner file but also an STL), and purchase and operating costs. Accuracy (precision and trueness) represents an important aspect to compare traditional and digital impressions, according to ISO 5725-1; the precision represents the degree of dispersion of different measurements from each other, whereby the higher the precision is, the more similar different measurements are [31]. The trueness describes the discrepancy between the measurement and the real dimension of the object. High trueness delivers a result that is close or equal to the actual dimensions of the measured object [6]. Compared to precision, trueness is harder to obtain since it requires the real object’s dimensions and simple geometries. Many in vitro [32,33,34,35] and few in vivo [1,9,36] studies were conducted on several impression materials and techniques with different results in terms of accuracy and precision of 3D impressions vs. traditional techniques; the works focused on single reconstructions, oral quadrants, and full dental arch. Lutharrdt et al. [37], found that the accuracy of a digital impression obtained using a Cerec 3D camera, is equal to 28 µm; Guth et al. [27] found that direct 3D digitization with LAVA showed higher accuracy compared to traditional methods. Mehl et al. [38] reported an accuracy of 19 µm for a Cerec AC Bluecam [39].

Recently, the validity of a digital model produced following an indirect method was evaluated in a systematic review by comparing digital and plaster models, thus concluding that digital models offer a high degree of validity [39,40]. Trueness measurements for conventional intraoral impressions with gypsum casts are most frequently done with linear distance measurements [41]. In general, two modes can be used to analyze the trueness and the precision of different impression systems: the first one consists of the comparison of digital files using commercial software, and the second consists of the comparison of the fit of restorations obtained with different systems. Regarding the comparative evaluation of digital files, in the literature, different methods were used to evaluate the accuracy of the digital impression, including the measure of surface points with high trueness using coordinate measuring machines (CMMs), but these lack in scan speed and do not accurately measure freeform surfaces because of the geometric size and shape of the tip (probe) [42,43,44,45]. Optical scanners with high accuracy are currently limited to the measurement of single teeth or quadrants [18,46]. Another way would be to compare the surface resulting from stereolithography (STL) datasets, whose function would be the input for CAD reconstruction. The 3D discrepancies between two surface datasets can be analyzed by superimposition using appropriate inspection software [10]. Any approach for the comparison of acquired geometries passes through the acquisition algorithms, which may be either direct (Procrustes analysis) or, more often, iterative (ICP, iterative closest point). In the bibliography, it is common to use commercial software to compare geometries, whether they come from measuring or mathematical approaches. The purpose of the present work is to evaluate the metrological performances of different 3D scanning systems, both desktop and intraoral, both with classical linear distance methodology and a volumetric error approach proposed by the authors. It has to be noted that the comparison of different intraoral scanning systems is very important for the clinician, among other, more obvious reasons, for choosing the most suitable materials for prosthetic reconstruction (ceramic, metal, zirconia, etc.). The null hypothesis is that there are no significant differences between the digital scanning systems using commercial software or the custom ICP technique.

## 2. Materials and Methods

### 2.1. Master Model Molding

The study did not involve human participants, specimens, or samples of vertebrate or vertebrate animals, embryos, or tissues. For this reason, the authors stated that the study did not involve human participants. For this reason, no ethics committee was requested. The authors declare that the full arch imprint obtained as a zirconia master model was acquired by a cast model of an author, granted for the scientific work. Starting from a cast model, an alginate imprint was obtained, and a reference zirconium model was produced. One of the authors with a complete dentition was recruited for a full arch imprinting using silicon material (Elite HD, Zhermack, Badia Polesine, Italy). The impressions were disinfected for 10 min (Impresept, 3M ESPE, Maplewood, NJ, USA) and poured in type IV dental stone (Moldastone CN, Heraeus Kulzer, Wasserburg am Bodensee, Germany).

A first inferior molar with normal anatomical features was obtained from the cast model and identified as the reference model. The choice of a molar was due to its complex morphological characteristics like different cusps, marginal ridges, pits, and fissures. The sixth molar selected was divided and scanned using an optical scanner D7000 (3Shape, Copenaghen, Denmark), obtaining an STL file. The file was processed by a CAM software connected to a milling machine (Zenotec T1, Zenotec limited, Tuam, Ireland). The model made of zirconium (Zenotec ZR Bridge) to ensure high hardness and stability over time was sectioned and milled. These features are fundamental for obtaining reliable measurements using contact measuring systems.

### 2.2. Reference Digitalization

To obtain a reference measurement of the master model, a coordinate measurement machine (CMM) was used to acquire high-accuracy point clouds of the master surface. The machine output is a point cloud describing the surface of the measured object; a feeler pin touching the surface of the object was used to acquire the points. The uncertainty of the CMM was 0.1 µm (Table 1).

At the end of the process, the geometry of the object can be reconstructed using a triangle-based rendering. After several measurements, it was possible to obtain the superficial geometry of the object with triangle-shaped modeling, obtaining an STL (standard triangulated language) file. Due to its high accuracy and high reliability, CMMs are often used in industrial applications for dimensional inspection. The CMM used in this study was manufactured by Brown&Sharpe DEA with a Renishaw touching probe. The metrological features are shown in Table 1.

### 2.3. Impression Scanning

To evaluate the effectiveness of the proposed methodology, four STL files were created by scanning the model with three desktop scanners: 3Shape D700 (3shape), 5Series (Dental Wing), and Sinergia Scan (Nobil-Metal), and one intraoral scanner: Trios (3Shape). Table 2 shows the different scanners and the abbreviations used in this work.

All scanners were not capable of providing any information on geometrical features close to the roughness scale; thus, any analysis close to the micrometric scale was not considered in this work [46,47,48,49,50].

### 2.4. Data Analysis with Commercial Software

The measurement performed with CMMs was taken as a reference for all the other comparisons. The comparisons were made using commercial software (Geomagic and 3-Matic, Carolina del Nord, NC, USA) and with custom algorithms developed in the MATLAB environment. Each STL file obtained from scans (STL format) was transformed into a point cloud for a comparison with the output of the CMM. The point clouds obtained were compared and analyzed with the ones acquired by two frequently used commercial software: Geomagic (Geomagic Control 2014, 3D Systems, Carolina del Nord, NC, USA) and 3-Matic (3-Matic Analyze-Mimics Innovation Suite, Materialise, Leuven, Belgium).

Geomagic is a set of products for CAD design, 3D scan management, and analytical and statistical inspection. Geomagic Control, part of the Geomagic Suite, is a metrology software platform that enables, among other things, to compare two or more elements providing statistical analysis of the comparison. Mimics Innovation Suite is a suite of biomedical engineering software, for segmentation processes and CAD design. Lastly, 3-Matic is a CAD suite that, apart from designing, allows the analysis and comparison of a couple of items, providing statistical analysis of such a comparison.

### 2.5. Data Analysis with Proposed Algorithm: MATLAB Environment

MATLAB is a programming language that allows the implementation of scripts for a variety of needs. The software also has a huge and constantly updated toolbox library; MATLAB is widely used in the academic world for processing data of any kind.

**Figure 1:**
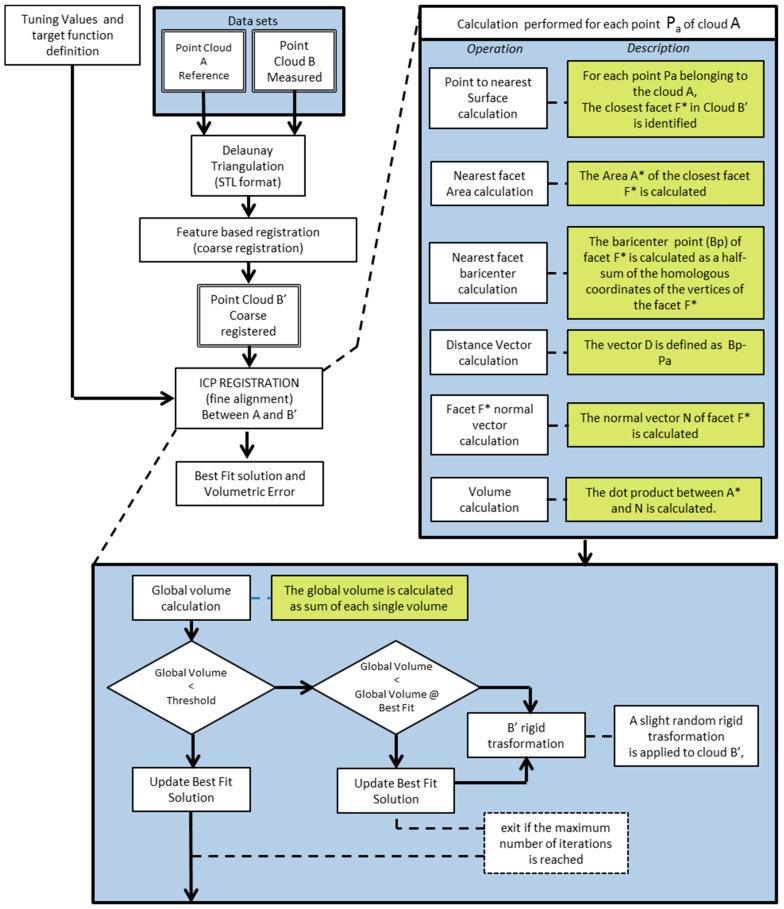
Architecture of the developed registration algorithm.

Each commercial software uses its own tuning set of algorithms for the registration, which leads to different results between them. The tuning of this control is in some cases difficult; to overcome this problem, a MATLAB code, developed by authors in a previous study, was applied to obtain complete control of the algorithms [51]. Specifically [52,53,54,55,56], the MATLAB code was based on registration algorithms using an iterative approach (ICP) similar to commercial software. Figure 1 describes the architecture of the developed registration algorithm. The algorithm improves the classic point-to-point ICP registration, introducing point-to-surface distance optimization; thus, the objective function is to minimize the volumetric error. In this case, a volumetric error is considered as the evaluator of the method, instead of the point-to-point distance calculated in commercial software.

This volumetric approach is a native three-dimensional description and can be used on point cloud datasets, like the traditional approach widely used in commercial programs.

### 2.6. Statistical Analysis

After the alignment of the two point clouds, the software that we used provided statistical information such as maximum, minimum, and mean of the distances and the related standard deviation. To verify the repeatability of the full process, a set of 30 measurements were performed for each scanner under analysis. In every measurement, the position of the measured item was changed.

According to the one-way ANOVA, the mean precision values are statistically different among the groups. The results of each acquisition, at *p* < 0.01, are detailed in Table 3. The level of significance was α = 0.05. In Table A1, Table A2, Table A3 and Table A4 the results of the statistical analysis over the entire population are reported (Appendix A).

## 3. Results

### 3.1. Comparison of Geometries with Commercial Software

**Figure 2:**
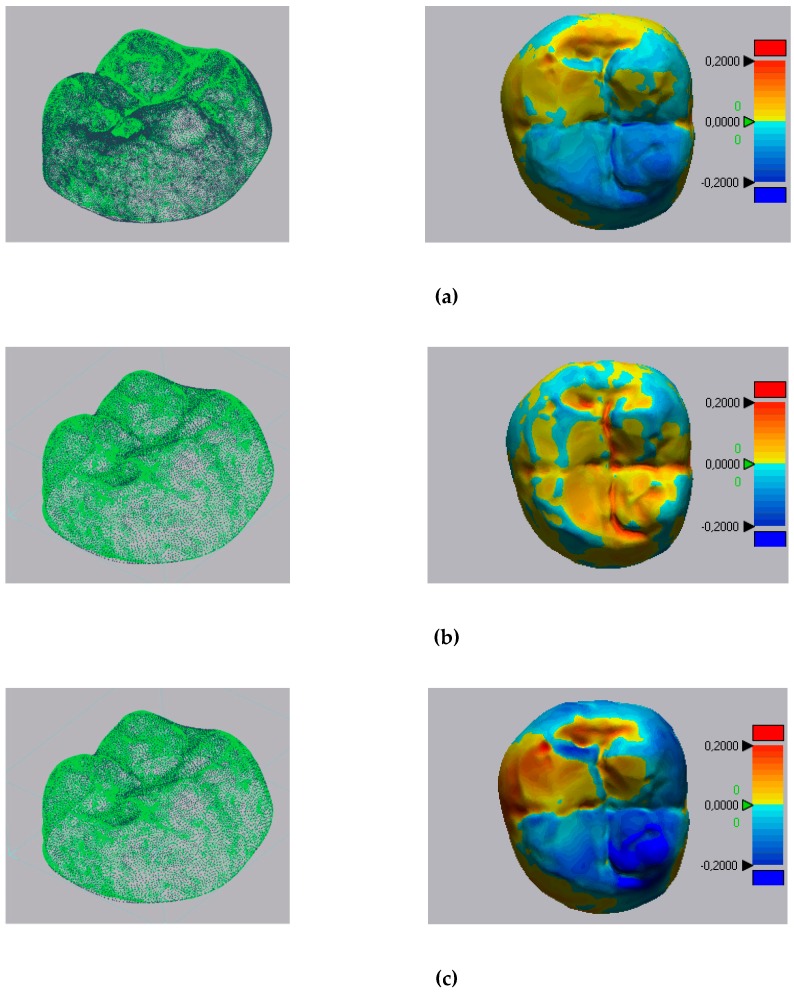

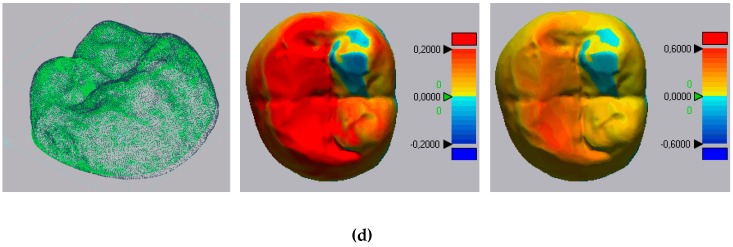
Point clouds registered and results of registrations with Geomagic: (**a**) D1 scanner; (**b**) D2 scanner; (**c**) D3 scanner; (**d**) I scanner. Results are expressed in mm. D—desktop; I—intraoral.

**Figure 3:**
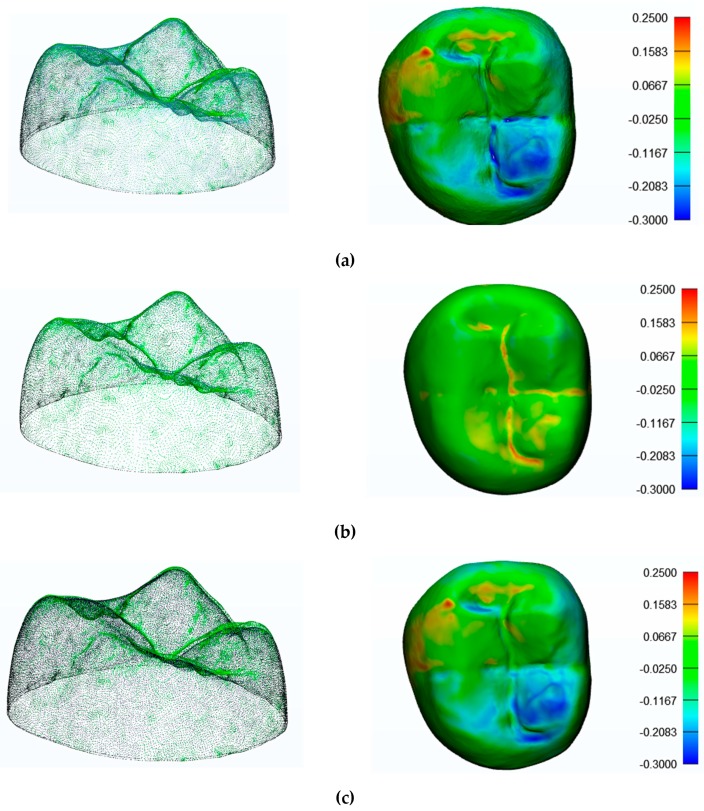

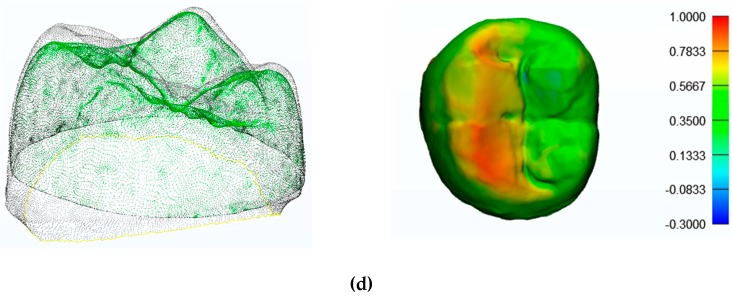
Point clouds registered and results of registrations with 3-Matic: (a) D1 scanner; (**b**) D2 scanner; (**c**) D3 scanner; (**d**) I scanner. Results are expressed in mm.

Figure 2 and Figure 3 show the results obtained with Geomagic and 3-Matic. Table 3 shows the results in terms of mean, maximum, and minimum values and standard deviation of distances between points laying on the compared geometries. Columns 1 and 2 report, respectively, the minimum and maximum values; column 3 shows the mean value of medians (when such medians are producible from the software). Columns 4 and 5 show, respectively, the means of the mean values deriving from every single measurement, and the mean values of their standard deviations. The 3-Matic software also gives also median values, but they cannot be compared with Geomagic’s values since this software does not provide these results. The mean values and standard deviations calculated with the Geomagic software were 0.0286 mm and 0.0551 mm for the D1 scanner, 0.0388 mm and 0.0428 mm for the D2 scanner, 0.0545 and 0.0863 for the D3 scanner, and 0.1654 mm and 0.1391 mm for the I scanner.

The mean values and standard deviations calculated with 3-Matic were −0.0396 and 0.094 for the D1 scanner, 0.025 and 0.0441 for the D2 scanner, −0.0269 and 0.0863 for the D3 scanner, and 0.1387 and 0.1303 for the I scanner (all values are expressed in millimeters).

### 3.2. Comparison of Geometries with MATLAB Custom Algorithm Results

**Figure 4:**
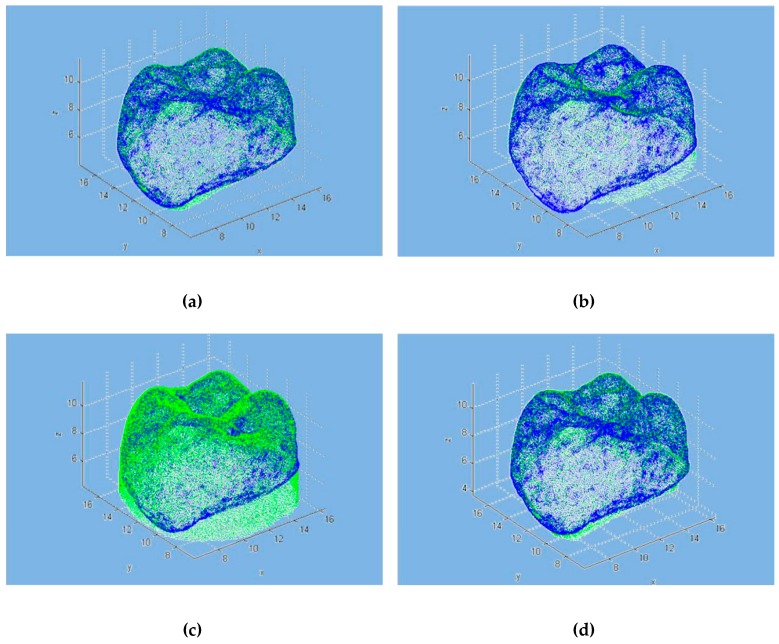
Point clouds of geometries with MATLAB: (**a**) D1 scanner; (**b**) D2 scanner; (**c**) D3 scanner; (**d**) I scanner. Data mapped are expressed in mm.

**Figure 5:**
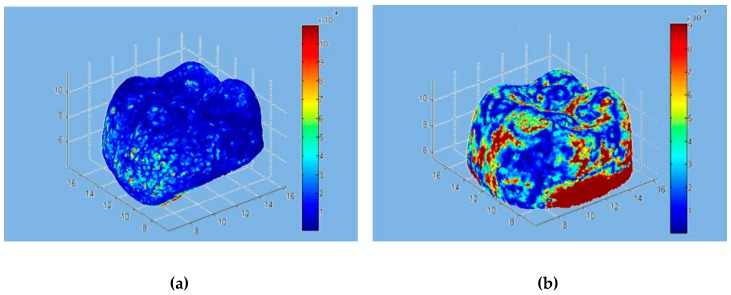

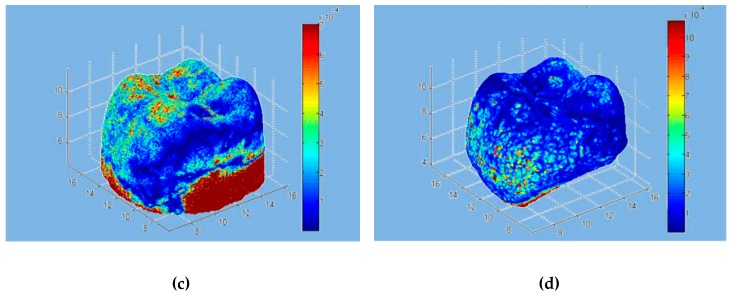
Volumetric errors with MATLAB: (**a**) D1 scanner; (**b**) D2 scanner; (**c**) D3 scanner; (**d**) I scanner. Results are expressed in mm^3^.

**Figure 6:**
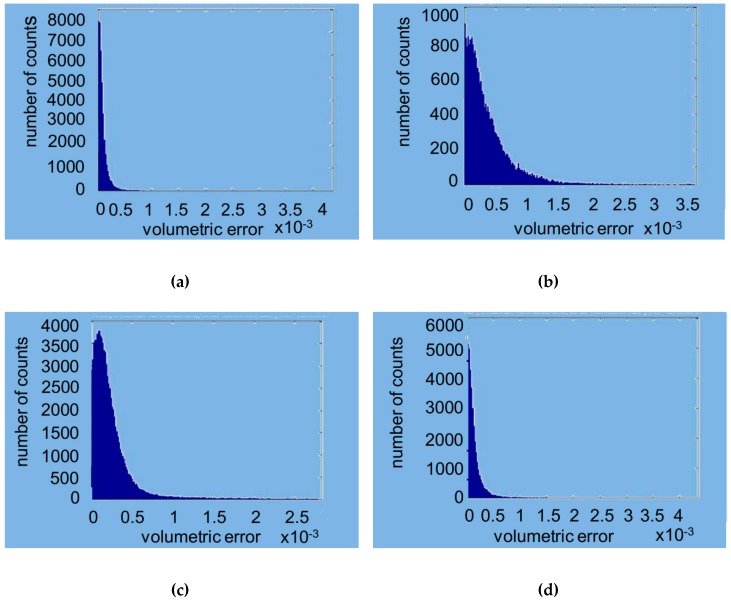
Volumetric errors distribution histograms with MATLAB: (**a**) D1 scanner; (**b**) D2 scanner; (**c**) D3 scanner; (**d**) I scanner. Volumetric error is expressed in mm^3^.

As with the commercial software approach, the model was superimposed on the individual scans using the developer tools. Figure 4 shows the overlapping points of clouds for all the scanners. Figure 5 represents the volumetric error calculated with the algorithm proposed, while, in Figure 6, the volumetric error distribution histograms are reported.

Table 4 shows the mean, median, and variance, and the minimum and maximum values of the volumetric errors.

**Figure 7:**
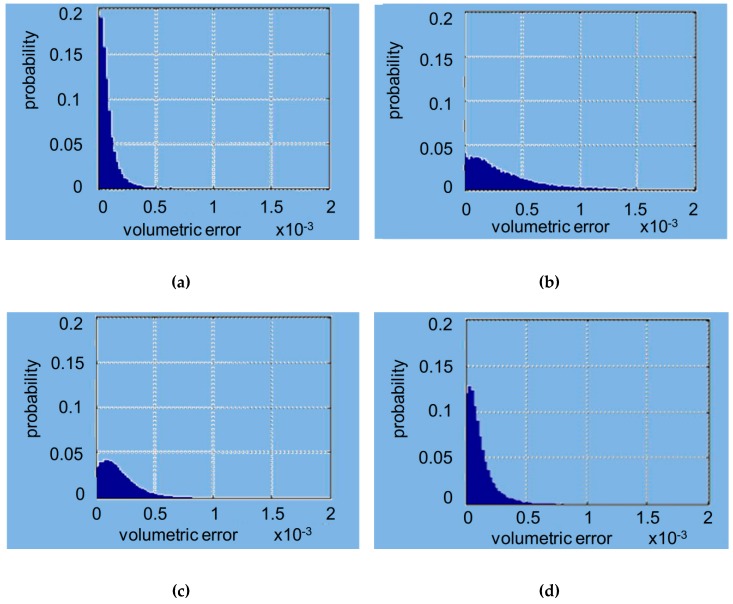
Probabilistic volumetric errors with MATLAB: (**a**) D1 scanner; (**b**) D2 scanner; (**c**) D3 scanner; (**d**) I scanner. Volumetric errors are expressed in mm^3^.

**Figure 8:**
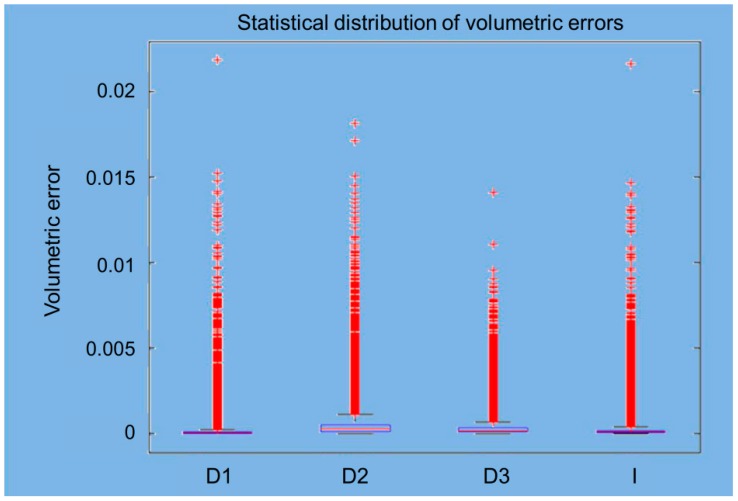
Box plots of volumetric errors with MATLAB: (**a**) D1 scanner; (**b**) D2 scanner; (**c**) D3 scanner; (**d**) I scanner. Volumetric errors are expressed in mm^3^.

The mean volumes and standard deviations calculated with MATLAB were 0.00014156 and 0.00052291 for the D1 scanner, 0.00049597 and 0.00091085 for the D2 scanner, 0.00031453 and 0.0005245 for the D3 scanner, and 0.00020287 and 0.00056518 for the I scanner (all values are defined in mm^3^). In Figure 7, the trends of the probabilistic volumetric errors for each used scanner are shown, while Figure 8 shows the box plots of the volumetric errors.

## 4. Discussion

The growing diffusion of CAD/CAM technologies in dentistry determined the need to obtain simple and reproducible methods to compare digital and conventional impression techniques. This research should start from natural tooth anatomy and from the scanner’s capability to capture its geometry, especially in prosthetic rehabilitation. In the last few years, this aspect was analyzed by using linear distance measurements, even if the results showed some variability mainly related to the operator’s ability [2,4,5,9,15,29,57,58,59,60].

In this work, a comparison between the metrological performance of different dental scanners was made, involving the linear distance measurements and volumetric error analysis. Several previous studies used commercial programs to determine the trueness of intraoral scanners. In this, however, an attempt was made to apply a volumetric error analysis to the dental field.

It is interesting to note that the maximum and minimum values of each measurement remain very close to the general mean, showing the absence of major errors in point coordinate measurements due to good characteristics of the measurement devices and to automatic corrections performed by the software while recreating the point clouds. Further investigations about this aspect have to be performed.

The mean values of the point-to-point distance remained of the same order for all three desktop scanners (D1, D2, D3), while we registered a significant worsening for the intraoral scanner (I).

The mean error measured with the I scanner was greater than the D scanners’ values with both techniques. The I scanner’s standard deviation was higher than the D scanners’ values, with a greater measurement dispersion. According to these results, the Metrologic I scanner’s performance was lower than the D ones. The results reject the null hypothesis of the study.

Generally, a three-dimensional scanner produces a non-uniform mapping of points in space. This means that each point acquired contributes within the same way for the length, but the volume error depends on the local density of the cloud, where a lower density corresponds to a higher error. The different programs provide users with different sizes and a direct comparison can be made only based on variables common to both programs. From the available data, it is not possible to define which program will outperform the other. For example, it can be noted that, while the spread between maximum and minimum values varies significantly, this leads in some cases to a substantial modification in the statistical distribution of the distances (D1 scanner), while, in other cases, there are no significant changes. Figure 4a shows the good alignment of the point clouds in the occlusal area, while Figure 4b–d show areas where the overlap is not optimal. This is clear in Figure 8, where there are many measuring points outside the limit. This is mostly due to the presence (in the different scans) of elements that have no direct correspondence on the point cloud generated by the CMM. This phenomenon is particularly evident in Figure 4b,c, where the measured points (in green) do not have a direct correspondence in the CMM scanning.

The software written in MATLAB looks for global optimization, i.e., it tries to find the best possible location even for the points that do not have a correspondence between the two scans, via the minimization of an objective function of the global type. The obtained effect is to introduce an error distributed over the entire volume of measurement. In place of the calculation of the point-to-point distances typically used in commercial software, the approach used by MATLAB aims to minimize point-to-surface distance, which implies the minimization of the volume between the registered areas. This was possible by introducing the metric for the calculation of volumes and using the minimization of volumes as to the objective function for the process of optimization of the registration phase. In the proposed approach, the volumes have to be considered, rather than the distances. Figure 5 shows, in a false color scale, the recording of volumetric errors. As expected, the major errors occur in the lower area of the model where the point clouds deriving from the CMM and the scanner may differ significantly. It is, however, to be noted that the greatest similarity of clouds relative to the scanners 1–4 leads to a lower overall volumetric error, clearly evident in Figure 5a, while Figure 5b,c show how the diversity of the clouds in the lower area constitutes a mistake of registration also in the coronal area. From Figure 5a, the values were 0.00014156 mm^3^ (SD 0.00052291) for the D1 scanner, 0,00049597 mm^3^ (SD 0.00091085) for the D2 scanner, 0.00031453 mm^3^ (SD 0.00052450) for the D3 scanner, and 0.00020287 mm^3^ (SD 0.0005618) for the I scanner. Figure 6 shows the histogram of the local volumetric error distribution. It is evident that the errors are concentrated in the low area abscissa, showing the substantial absence of a bias error. Figure 7 compares the volumetric error probability distributions for measurements performed with the different scanners. For this distribution, it can be observed that D1 and I scanners have a low probability of errors with respect to D2 and D3 scanners.

Using comparative analysis based on linear analysis and volumetric error analysis allows us to obtain an evaluation of the performances of the scanners [61]. In particular, the evaluation based on linear distances deriving from the commercial software shows a lower metrological performance compared to desktop ones. The additional comparison performed with MATLAB demonstrated the same behavior as well, but a direct comparison between the two approaches cannot be easily applied and deserves further analysis.

The limitation of this study is that some investigations must be done regarding the full arch in terms of volumetric analysis.

## 5. Conclusions

The following conclusions were drawn: all of the digital impression systems were able to measure the specific tooth structure; each tested system showed different levels of trueness and precision values; commercial programs are reliable methods to analyze accuracy and precision; a different approach based on volumetric error calculation was proposed in addition to classical linear error calculation; the results seem to demonstrate the good reliability of the procedure.

Further studies to validate the volumetric error approach are necessary, as well as its extension to full arch data acquisition.

## Figures and Tables

**Table 1:** Coordinate measurement machine (CMM) characteristics.

**Manufacturer**	Brown&Sharpe DEA S.p.a.
**Model**	Scirocco MP101509
**Performance Compliance**	ISO 10360-2
**Calibration Certificate date**	2016-04-29
**Measure Volume**	1000 × 1500 × 2012 (mm)
**Head Type**	Renishaw PH10MQ
**Gauge**	Renishaw TP20
**Polar radius max difference (25 measures)**	2.8 (μm)
**Uncertainty (K = 2), up to**	0.1 (μm)
**MPE P**	3.5 ± 1.75 (μm)
**Gauge error**	2.8 (μm)

**Table 2:** The different scanners used.

Abbreviation	Name	Manufacturer	Accuracy	Photo
**D1**	3Shape D700	3Shape	20 µm	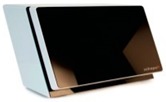
**D2**	5Series	Dental Wing	20 µm	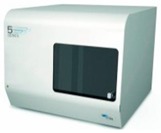
**D3**	Sinergia Scan	Nobil-Metal	12 µm	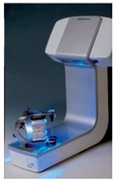
**I**	Trios	3Shape	20 µm	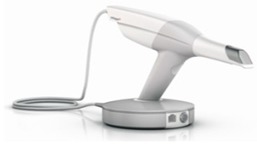

**Table 3:** Mean, maximum, minimum, and standard deviation values of compared geometries with commercial software. Values are calculated as mean values among the entire population of data. D—desktop; I—intraoral.

Scanner	Software	Min	Max	Median	Mean	SD
		(mm)	(mm)	(mm)	(mm)	(mm)
**D1**	3-Matic	−0.3103	0.0321	−0.0373	−0.0396	0.094
	Geomagic	−0.4122	0.3062	---	0.0286	0.0551
**D2**	3-Matic	−0.1018	0.2624	0.0187	0.025	0.0441
	Geomagic	−0.0239	0.2579	---	0.0388	0.0428
**D3**	3-Matic	−0.2906	0.2172	−0.0196	−0.0269	0.0863
	Geomagic	−0.2906	0.2221	---	0.0545	0.0863
**I**	3-Matic	−0.1285	0.5273	0.1134	0.1387	0.1303
	Geomagic	−0.4505	0.5273	---	0.1654	0.1391

**Table 4:** Mean, median, and variance, and the minimum and maximum volumes of the volumetric errors of compared geometries with MATLAB.

Scanner	Mean (mm^3^)	Median (mm^3^)	STD (mm^3^)	Min (mm^3^)	Max (mm^3^)
**D1**	1.4156 × 10^−4^	6.0725 × 10^−5^	5.2291 × 10^−4^	2.8328 × 10^−10^	0.0219
**D2**	4.9597 × 10^−4^	2.7183 × 10^−4^	9.1085 × 10^−4^	1.1154 × 10^−8^	0.0182
**D3**	3.1453 × 10^−4^	1.8008 × 10^−4^	5.2450 × 10^−4^	1.1310 × 10^−9^	0.0141
**I**	2.0287 × 10^−4^	9.2062 × 10^−5^	5.6518 × 10^−4^	1.5876 × 10^−9^	0.0217

## Appendix A

**Table A1:** Scanner D1: results of the statistical analysis over the entire population. Mean, maximum, minimum, and standard deviation values of compared geometries with commercial software.

Sample n°	Scanner D1: Min [mm] 3-matic	Scanner D1: Max [mm] 3-matic	Scanner D1: Median [mm] 3-matic	Scanner D1: Mean [mm] 3-matic	Scanner D1: SD[mm] 3-matic	Scanner D1: Min [mm] Geomagic	Scanner D1: Max [mm] Geomagic	Scanner D1: Mean [mm] Geomagic	Scanner D1: SD [mm] Geomagic
1	−0.2885	0.0115	−0.0628	−0.0363	0.0814	−0.38545406	0.276725124	−0.04893728	0.055600599
2	−0.26876	0.0252	−0.0405	−0.0532	0.1083	−0.37093595	0.303139111	−0.01996574	0.044769622
3	−0.30531	0.019	−0.0617	0.0029	0.0981	−0.37368288	0.289543464	−0.00173811	0.073996361
4	−0.30372	0.008	−0.0467	−0.0278	0.0851	−0.4017169	0.286267097	−0.06426665	0.051998538
5	−0.30722	0.0006	−0.0458	−0.0443	0.102	−0.40603246	0.265953915	−0.06816115	0.062262669
6	−0.27031	0.0252	−0.0288	−0.0686	0.0864	−0.37767468	0.28051937	−0.0160112	0.070223043
7	−0.28462	0.0008	−0.0745	−0.0402	0.0781	−0.38332871	0.276554047	−0.02353725	0.072008235
8	−0.27174	0.0003	−0.0325	−0.0136	0.1044	−0.39164583	0.294442145	−0.04605321	0.045416321
9	−0.30609	0.0237	−0.0483	−0.0666	0.0815	−0.37024251	0.302135949	−0.06132818	0.04543797
10	−0.3042	0.0299	−0.0229	−0.0067	0.1103	−0.40867947	0.290691141	−0.05508965	0.066147463
11	−0.27261	0.0081	−0.0545	−0.0445	0.0814	−0.3786655	0.297845856	−0.01645955	0.063384042
12	−0.30993	0.0206	−0.0541	−0.0483	0.0868	−0.36256436	0.274582175	−0.00504935	0.056942696
13	−0.29971	0.0237	−0.0324	−0.0335	0.0916	−0.40549641	0.259519921	−0.04622169	0.065123292
14	−0.30771	0.0235	−0.0116	−0.0459	0.096	−0.38237552	0.259110778	−0.00326103	0.040148547
15	−0.29877	0.0068	−0.0168	−0.0033	0.1019	−0.40460144	0.284267483	−0.00384671	0.049719942
16	−0.27969	0.0226	−0.0351	−0.0536	0.084	−0.40771983	0.29649729	−0.02071697	0.068142639
17	−0.27361	0.0211	−0.0151	−0.0618	0.1064	−0.39129371	0.285217503	−0.00786584	0.045105455
18	−0.29876	0.0262	−0.0116	−0.0261	0.1096	−0.37816782	0.260653735	−0.00561344	0.045626003
19	−0.2781	0.0014	−0.0001	−0.0647	0.0959	−0.40477652	0.30451627	−0.02658745	0.051661294
20	−0.27688	0.0098	−0.0346	−0.0232	0.0772	−0.37010547	0.271668893	0.00190503	0.050605688
21	−0.2784	0.0169	−0.0271	−0.04	0.0924	−0.3901043	0.272363344	−0.02061957	0.06297591
22	−0.26954	0.0215	−0.0086	−0.0408	0.0955	−0.4023326	0.302295757	−0.06862174	0.042297196
23	−0.26298	0.0077	−0.0721	−0.0452	0.09	−0.39514631	0.297153096	−0.01107715	0.062633413
24	−0.26034	0.0019	−0.024	−0.048	0.1023	−0.39656539	0.289381906	−0.0030611	0.048203578
25	−0.27528	0.0066	−0.0705	−0.0661	0.0996	−0.39286144	0.258569199	−0.02369157	0.044807161
26	−0.29037	0.0226	−0.0683	−0.0687	0.0902	−0.37867023	0.300837065	−0.03693786	0.057959913
27	−0.29019	0.0147	−0.0633	−0.0115	0.0999	−0.40914412	0.284389628	−0.06022517	0.053896628
28	−0.28717	0.0061	−0.0156	−0.0713	0.082	−0.40814553	0.301813925	−0.03036833	0.050702256
29	−0.2681	0.0209	−0.0036	−0.0123	0.0964	−0.40996617	0.275787637	−0.03803396	0.057036145
30	−0.27895	0.0008	−0.0375	−0.0245	0.1087	−0.37012382	0.288388967	−0.02655814	0.048167383

**Table A2:** Scanner D2: results of the statistical analysis over the entire population. Mean, maximum, minimum, and standard deviation values of compared geometries with commercial software.

Sample n°	Scanner D2: Min [mm] 3-matic	Scanner D2: Max [mm] 3-matic	Scanner D2: Median [mm] 3-matic	Scanner D2: Mean [mm] 3-matic	Scanner D2: SD [mm] 3-matic	Scanner D2: Min [mm] Geomagic	Scanner D2: Max [mm] Geomagic	Scanner D2: Mean [mm] Geomagic	Scanner D2: SD [mm] Geomagic
1	−0.05559809	0.234735985	0.005188452	0.015044861	0.040011603	−0.016596856	0.239030035	0.044568789	0.039483152
2	−0.079878997	0.2234752	−0.029041417	−0.00533495	0.038541149	0.006775945	0.238065342	0.036471998	0.054091924
3	−0.094650934	0.235459432	−0.038287262	0.057053765	0.054767438	0.002674579	0.213585298	0.04597167	0.032883445
4	−0.082978553	0.234895242	−0.036483554	0.018257273	0.05357025	0.013187224	0.240011775	0.073075048	0.057173863
5	−0.089290001	0.242043471	−0.034817528	0.055937677	0.051643043	0.02241958	0.212302958	0.053286243	0.037526151
6	−0.094196344	0.233567315	0.002372854	0.044651262	0.031525171	−0.019191686	0.21550202	0.03024454	0.040416117
7	−0.08665247	0.253418581	−0.053414435	0.003382346	0.059631522	0.008584724	0.242304096	0.029611291	0.033038573
8	−0.07803046	0.250341111	−0.037287012	0.040198773	0.036962065	0.005213882	0.212859701	0.035924326	0.050874009
9	−0.071364294	0.259518304	−0.023500462	0.055674948	0.051957324	−0.010659493	0.237952216	0.001938023	0.048503503
10	−0.077967423	0.234525853	−0.05272095	0.051754075	0.040672267	0.024751507	0.231409636	0.036502749	0.0443001
11	−0.074460155	0.261552656	−0.049905676	0.02122489	0.054119402	−0.019955628	0.219989993	0.02449097	0.026033484
12	−0.093435767	0.219585312	0.015092024	0.010846666	0.030194845	−0.009175737	0.237299049	0.026956223	0.046847739
13	−0.095493586	0.239723286	−0.039745544	0.031874569	0.047589366	0.019569632	0.230508414	0.039900278	0.024481113
14	−0.087406718	0.244379261	−0.043395828	0.00233264	0.0278432	0.007200076	0.239544986	0.039312111	0.05235953
15	−0.09316826	0.222852287	−0.026583558	−0.009644497	0.037603005	−0.02147101	0.246618525	0.028449294	0.037169703
16	−0.063558113	0.220461847	−0.046924348	0.050575846	0.044434621	−0.006206507	0.213522839	0.055873168	0.047579536
17	−0.099786947	0.240640095	−0.040210836	0.044004416	0.058841372	0.002862416	0.247935963	0.044382685	0.032934142
18	−0.09571366	0.231879772	−0.049921057	0.032762332	0.058688293	−0.00161732	0.214176942	0.065488819	0.046906082
19	−0.096496409	0.2524543	-6.63E-05	0.055600465	0.02647297	0.021941952	0.249702482	0.02850467	0.05763061
20	−0.056612949	0.252046073	−0.000203084	−0.007344192	0.055170739	0.004669284	0.225967725	0.059806344	0.043873914
21	−0.068318089	0.245444284	0.001477482	0.053230343	0.05357518	−0.006495504	0.22170474	0.002601997	0.047615307
22	−0.078092437	0.235603597	0.012484246	0.048197805	0.035767248	−0.000516601	0.221063933	0.068867886	0.053460028
23	−0.059482741	0.249081672	−0.052432452	−0.009648531	0.04656202	−0.021088249	0.215532105	0.020729887	0.047664035
24	−0.082352509	0.247768547	−0.044205592	−0.007854655	0.03078478	0.009957894	0.21222616	0.074483778	0.057447131
25	−0.0828698	0.250334262	0.007473731	0.002004107	0.058578741	−0.005851656	0.231746344	0.040073564	0.02512131
26	−0.086593431	0.245878227	−0.055776121	0.021900566	0.0334415	0.005638017	0.218707713	0.048717413	0.030059703
27	−0.07075342	0.22330662	−0.024378255	0.011410853	0.031208516	−0.0017974	0.247131175	0.025584927	0.039479181
28	−0.098065754	0.214475158	0.001400795	0.055925483	0.037787586	0.001404241	0.207987499	0.029776333	0.056168042
29	−0.097681818	0.219984572	−0.054021264	−0.013020418	0.037781806	0.002768872	0.217710293	0.019367593	0.033486409
30	−0.070790562	0.251131126	−0.02880334	0.019001283	0.057272978	0.002772199	0.255505325	0.033037383	0.039392164

**Table A3:** Scanner D3: results of the statistical analysis over the entire population. Mean, maximum, minimum, and standard deviation values of compared geometries with commercial software.

Sample n°	Scanner D3: Min [mm] 3-matic	Scanner D3: Max [mm] 3-matic	Scanner D3: Median [mm] 3-matic	Scanner D3: Mean [mm] 3-matic	Scanner D3: SD [mm] 3-matic	Scanner D3: Min [mm] Geomagic	Scanner D3: Max [mm] Geomagic	Scanner D3: Mean [mm] Geomagic	Scanner D3: SD [mm] Geomagic
1	−0.254310217	0.178069565	0.01541129	−0.028719584	0.079945746	−0.264776546	0.184517562	−0.033473335	0.097442048
2	−0.245332214	0.186576515	−0.055368173	−0.05046097	0.094199931	−0.256215369	0.195760877	−0.046362529	0.0859752
3	−0.276714554	0.2082844	−0.042906972	−0.023604206	0.073481657	−0.270589633	0.185760442	−0.027473323	0.084159645
4	−0.262810076	0.195895677	0.005396755	−0.05588251	0.098131013	−0.282996778	0.204212025	−0.076685213	0.073126555
5	−0.290327668	0.175718849	0.002924972	0.003700348	0.090169938	−0.286862586	0.184893945	−0.052759724	0.082404603
6	−0.29011559	0.192048751	0.011518932	−0.056650825	0.099730572	−0.244755377	0.194496313	−0.030458023	0.089906971
7	−0.266263505	0.212237894	−0.011439302	−0.03482762	0.093784678	−0.269797927	0.18993196	−0.049143057	0.088848151
8	−0.244765509	0.198089411	−0.007124041	−0.043516498	0.080398107	−0.266227758	0.193729547	−0.027826013	0.103418378
9	−0.244104377	0.195664308	−0.023080695	−0.05007469	0.090107952	−0.267912775	0.178157403	−0.090768961	0.079294486
10	−0.252607625	0.202303083	−0.019095623	−0.05475524	0.086065576	−0.252942313	0.182666535	−0.092989839	0.073845791
11	−0.280103874	0.176313915	−0.016119346	−0.059788134	0.079087947	−0.261700778	0.17851878	−0.045311494	0.092746223
12	−0.289976795	0.177175083	−0.023622587	0.003828915	0.097145488	−0.256864302	0.209846693	−0.052254141	0.084507914
13	−0.278218574	0.212038672	−0.003946003	−0.049882344	0.083915774	−0.251686565	0.196755571	−0.093406448	0.071999315
14	−0.264012588	0.175074466	0.014270096	0.008349977	0.10006406	−0.281608359	0.221303289	−0.028943935	0.087137103
15	−0.242468328	0.187963485	−0.008963086	−0.009603513	0.070863165	−0.288910445	0.184370625	−0.041414969	0.103385624
16	−0.289198419	0.173462374	0.017668911	−0.018196157	0.076888822	−0.242950347	0.184033623	−0.045536777	0.101012178
17	−0.243604655	0.210867521	−0.00027629	−0.000231042	0.081336703	−0.245736532	0.181974381	−0.07902084	0.078338864
18	−0.270019685	0.201494977	−0.035651628	−0.037627209	0.0792426	−0.262892094	0.205339708	−0.051040684	0.080077924
19	−0.244814798	0.209215056	0.009522518	−0.047046718	0.076371406	−0.247015252	0.202492287	−0.09656671	0.092188097
20	−0.254331832	0.20604674	−0.002933949	−0.049728139	0.098762815	−0.288111452	0.177005765	−0.026781972	0.091714406
21	−0.243574319	0.205360454	0.014191011	−0.026155182	0.069495636	−0.268968144	0.196514392	−0.072433713	0.071350331
22	−0.287046597	0.178264631	−0.011170269	−0.00437325	0.07261242	−0.254672641	0.185874091	−0.039114598	0.096651297
23	−0.258481863	0.181437936	0.010469901	−0.042052681	0.088648845	−0.287646237	0.184605187	−0.092939708	0.08174602
24	−0.285257641	0.211889049	−0.044839747	−0.036283616	0.082438987	−0.279499543	0.204800837	−0.053647486	0.091646069
25	−0.280475922	0.209508775	−0.028061188	−0.025497988	0.091810286	−0.276645669	0.208593129	−0.030006671	0.093939263
26	−0.256539612	0.17959888	−0.051818611	−0.0067587	0.081766499	−0.26493827	0.207908965	−0.044338572	0.100260316
27	−0.25762658	0.183942233	−0.000991685	−0.02199195	0.091106159	−0.280490756	0.201153956	−0.046400184	0.080933861
28	−0.26738281	0.206320205	−0.00194047	0.001218856	0.096626083	−0.263384327	0.202752533	−0.043634908	0.071905164
29	−0.274765893	0.174749396	−0.018205667	−0.000392787	0.085241639	−0.275289398	0.194538021	−0.041025597	0.076496789
30	−0.27994523	0.183664002	−0.000599737	0.010003458	0.099559494	−0.257619673	0.187234597	−0.083240573	0.082541416

**Table A4:** Scanner I: results of the statistical analysis over the entire population. Mean, maximum, minimum, and standard deviation values of compared geometries with commercial software.

Sample n°	Scanner I: Min [mm] 3-matic	Scanner I: Max [mm] 3-matic	Scanner I: Median [mm] 3-matic	Scanner I: Mean [mm] 3-matic	Scanner I: SD [mm] 3-matic	Scanner I: Min [mm] Geomagic	Scanner I: Max [mm] Geomagic	Scanner I: Mean [mm] Geomagic	Scanner I: SD [mm] Geomagic
1	−0.11781247	0.49795063	0.008689796	−0.1742938	0.13664737	−0.4222614	0.51627399	−0.145478598	0.152366066
2	−0.124892019	0.51138728	0.021866352	−0.1636303	0.1347985	−0.4442221	0.51579094	−0.163867642	0.145192668
3	−0.097546755	0.49686383	−0.01183871	−0.1128749	0.14219212	−0.4098171	0.50704209	−0.149092445	0.144715937
4	−0.102007814	0.49035466	−0.01395825	−0.1326804	0.12855203	−0.4269133	0.5219809	−0.148277286	0.137446306
5	−0.089735162	0.51364537	−0.02998977	−0.1265968	0.14216613	−0.4340189	0.52117973	−0.19305626	0.134018058
6	−0.119371988	0.48490691	−0.01333214	−0.1053726	0.1216599	−0.4141755	0.47849145	−0.202578541	0.155360827
7	−0.092763599	0.51238044	0.012373462	−0.1106396	0.14059593	−0.4102756	0.4961868	−0.153089979	0.153375816
8	−0.079816584	0.51509974	0.017380729	−0.1474741	0.12697789	−0.4227497	0.50512709	−0.186467778	0.13828498
9	−0.125690003	0.49006616	0.007894965	−0.1104756	0.13145195	−0.4501583	0.51281377	−0.169186181	0.148139641
10	−0.096790949	0.50712252	−0.04491549	−0.105626	0.12507113	−0.449114	0.50858314	−0.173226218	0.141503619
11	−0.08176709	0.48380357	−0.04712203	−0.1161692	0.12018203	−0.4412199	0.51835278	−0.203100171	0.135762015
12	−0.08942935	0.47777387	0.024462985	−0.1736686	0.1288828	−0.4254483	0.48049166	−0.161042323	0.121542016
13	−0.114175134	0.52680922	−0.00702429	−0.127794	0.12556558	−0.4458986	0.49690159	−0.15511584	0.13261246
14	−0.097592391	0.48457342	−0.0003491	−0.1432005	0.12228327	−0.4213368	0.50501752	−0.180934356	0.132151445
15	−0.126768593	0.49662327	−0.04227782	−0.1393851	0.13582834	−0.4447803	0.49978796	−0.203218517	0.149085868
16	−0.12625199	0.50752249	−0.03558122	−0.1617198	0.14511671	−0.4146253	0.50845198	−0.137539877	0.123564262
17	−0.116292692	0.52012804	−0.03972424	−0.1488023	0.14661289	−0.4010466	0.51320049	−0.138306097	0.122428148
18	−0.109952501	0.51338727	0.008726925	−0.1144032	0.11527645	−0.4269293	0.51804156	−0.1425405	0.130833686
19	−0.081442505	0.51454857	0.020090463	−0.1488778	0.13765427	−0.4315004	0.51842852	−0.173506192	0.136569566
20	−0.092419796	0.48527664	−0.00874486	−0.1128613	0.13112836	−0.4049787	0.50856657	−0.147672306	0.135382201
21	−0.124709961	0.48698669	0.017546582	−0.1220959	0.12597153	−0.4293716	0.51436094	−0.161701094	0.133814418
22	−0.099202757	0.48861968	−0.02268056	−0.1537021	0.14577961	−0.4286044	0.51825091	−0.153697755	0.122291941
23	−0.12453533	0.48328769	−0.00930665	−0.1718808	0.1404617	−0.4365059	0.48791528	−0.140175002	0.129642043
24	−0.124041271	0.4952397	−0.02401531	−0.1740354	0.12114903	−0.4194405	0.49181124	−0.186247028	0.147610025
25	−0.091844597	0.48432472	0.020236357	−0.1154929	0.11427098	−0.4499926	0.51558894	−0.168594521	0.150766974
26	−0.080288816	0.51846243	0.021326592	−0.1669572	0.11691116	−0.4244679	0.48474799	−0.168481123	0.125331508
27	−0.098828131	0.51264328	−0.00372945	−0.1301353	0.14128072	−0.4328578	0.5013414	−0.145632043	0.147953087
28	−0.089001162	0.52392736	−0.01411654	−0.1584058	0.12239955	−0.4207694	0.52583438	−0.159709246	0.151355366
29	−0.085326312	0.50104407	−0.03725311	−0.1406584	0.12426522	−0.4274239	0.51303678	−0.181544201	0.143912632
30	−0.115538035	0.48881803	0.005549855	−0.1510905	0.11786685	−0.4007188	0.51336581	−0.168920878	0.149986421
